# Transmission patterns of rifampicin resistant *Mycobacterium tuberculosis* complex strains in Cameroon: a genomic epidemiological study

**DOI:** 10.1186/s12879-021-06593-8

**Published:** 2021-08-31

**Authors:** Matthias Merker, Nkongho F. Egbe, Yannick R. Ngangue, Comfort Vuchas, Thomas A. Kohl, Viola Dreyer, Christopher Kuaban, Jürgen Noeske, Stefan Niemann, Melissa S. Sander

**Affiliations:** 1 Molecular and Experimental Mycobacteriology, Research Center Borstel, Borstel, Germany; 2 Evolution of the Resistome, Research Center Borstel, Borstel, Germany; 3 German Center for Infection Research, Partner Site Hamburg-Lübeck-Borstel-Riems, Borstel, Germany; 4 Tuberculosis Reference Laboratory Bamenda, Center for Health Promotion and Research, Bamenda, Cameroon; 5 School of Life Sciences, College of Science, University of Lincoln, Lincoln, England UK; 6 Faculty of Health Sciences, University of Bamenda, Bamenda, Cameroon; 7 Bamenda, Cameroon

**Keywords:** MDR-TB, *Mycobacterium tuberculosis*, Cameroon, Transmission

## Abstract

**Background:**

Determining factors affecting the transmission of rifampicin (RR) and multidrug-resistant (MDR) *Mycobacterium tuberculosis* complex strains under standardized tuberculosis (TB) treatment is key to control TB and prevent the evolution of drug resistance.

**Methods:**

We combined bacterial whole genome sequencing (WGS) and epidemiological investigations for 37% (n = 195) of all RR/MDR-TB patients in Cameroon (2012–2015) to identify factors associated with recent transmission.

**Results:**

Patients infected with a strain resistant to high-dose isoniazid, and ethambutol had 7.4 (95% CI 2.6–21.4), and 2.4 (95% CI 1.2–4.8) times increased odds of being in a WGS-cluster, a surrogate for recent transmission. Furthermore, age between 30 and 50 was positively correlated with recent transmission (adjusted OR 3.8, 95% CI 1.3–11.4). We found high drug-resistance proportions against three drugs used in the short standardized MDR-TB regimen in Cameroon, i.e. high-dose isoniazid (77.4%), ethambutol (56.9%), and pyrazinamide (43.1%). Virtually all strains were susceptible to fluoroquinolones, kanamycin, and clofazimine, and treatment outcomes were mostly favourable (87.5%).

**Conclusion:**

Pre-existing resistance to high-dose isoniazid, and ethambutol is associated with recent transmission of RR/MDR strains in our study. A possible contributing factor for this observation is the absence of universal drug susceptibility testing in Cameroon, likely resulting in prolonged exposure of new RR/MDR-TB patients to sub-optimal or failing first-line drug regimens.

**Supplementary Information:**

The online version contains supplementary material available at 10.1186/s12879-021-06593-8.

## Background

An estimated half a million tuberculosis (TB) patients are eligible for a rifampicin resistant or multidrug resistant (additional resistance to isoniazid) tuberculosis (RR/MDR-TB) treatment regimen each year [1]. To reduce the exceptionally long MDR-TB treatment period (minimum 18 months), high treatment costs, severe drug-related side effects and thus suboptimal adherence and low cure rates in many settings worldwide, in 2016 the WHO endorsed a shorter (9–12 months), standardized MDR-TB treatment regimen, including seven antibiotics. In the intensive phase (4–6 months) patients receive high dose isoniazid, ethambutol, pyrazinamide, prothionamide, clofazimine, kanamycin, and a fluoroquinolone. The 5 month continuation phase includes ethambutol, pyrazinamide, a fluoroquinolone, and clofazimine [2, 3]. The short MDR-TB regimen is recommend for patients infected with a RR/MDR *Mycobacterium tuberculosis* complex (MTBC) strain who have not been treated for more than 1 month with the above mentioned antibiotics and for whom resistance to fluoroquinolones and second-line injectable drugs has been excluded [4]. The most recent guidance is for a similar shorter, all-oral 9–12 month regimen with bedaquiline replacing the second-line injectable [5].

Notably, the short course, standardized MDR-TB regimen achieved high cure rates (> 85%) in Bangladesh, Cameroon, and Niger, however, its implementation in settings with high pyrazinamide and ethambutol resistance rates, e.g. in Eastern Europe, was criticized [6–9]. Prudent usage with careful follow-up has been recommended when resistance to isoniazid, pyrazinamide or second-line injectable drugs is present at the initiation of the short MDR-TB therapy [10–12]. Recent studies have shown that resistance to individual drugs, except fluoroquinolones, at the start of the therapy can be tolerated and did not compromise treatment outcomes [2, 10, 11]. More pronounced effects on treatment outcomes were observed when resistance to both a fluoroquinolone and pyrazinamide were diagnosed at baseline [2]. Besides the elevated risks of treatment failure under standardized treatment regimens, pre-existing drug resistances have been linked to increased transmission rates of MDR MTBC strains globally [13–15].

In Cameroon, a previous observational prospective study of patients treated between 2008 and 2011 reported 134/150 (89%) patients with successful outcomes on the short MDR-TB regimen [16]. Here, we analysed RR/MDR strains consecutively sampled from 195 patients between 2012 and 2015 at the Tuberculosis Reference Laboratory Bamenda (TBRL), representing 37.0% (195/527) of all laboratory confirmed MDR-TB cases in Cameroon during the study period (WHO data repository). We employed whole genome sequencing (WGS) and epidemiological analysis to define drug resistance profiles prior to the initiation of the short MDR-TB therapy, to investigate drug resistance proportions over time, and to identify factors associated with recent transmission of MDR-MTBC strains in Cameroon.

## Methods

### Study design and study population

We performed a retrospective genomic epidemiological study to analyse transmission patterns and risk factors for the transmission of RR/MDR MTBC strains in Cameroon. Specimens from consecutive TB-patients with bacteriologically-confirmed RR/MDR-TB received between December 31, 2011 and June 26, 2015 at the Tuberculosis Reference Laboratory Bamenda (TBRL) were included in the study. This laboratory serves as the reference laboratory for four geographical regions of Cameroon, covering an estimated population of approximately 7.8 million people and including 40% of the notified TB cases in the country from 2012 to 2015 (WHO data). Following the National TB Program guidelines, TB culture and drug susceptibility testing is performed systematically for previously treated TB patients, prisoners and known contacts of people with MDR-TB. Initial detection of TB disease in Cameroon was performed primarily with acid fast bacilli smear microscopy during the study period. For people initiating TB treatment with smear-positive TB, cure was defined as a negative smear at 5 and/or 6 months on treatment, following the NTP guidance. Further details on the data collection and contact tracing questionnaires are provided in the Additional file 1.

### Phenotypic and molecular drug susceptibility tests

Routine diagnostics were performed at the TBRL Bamenda, a laboratory accredited in accordance with the recognized International Standard ISO 15189:2012 (SANAS Accredited Medical Laboratory, No. M0593). Molecular tests for rifampicin resistance were performed either by Xpert MTB/RIF (Cepheid, U.S.) or Genotype MTBDR*plus* (Hain Life Science, Germany) according to the manufacturer’s instructions. The proportion method on Löwenstein–Jensen media with WHO recommended critical concentrations was employed for phenotypic drug susceptibility tests. Further details are provided in the Additional file 1.

### Next generation sequencing

WGS was performed on an Illumina NextSeq 500 instrument using Nextera XT library preparation kit according to manufacturer’s instructions (Illumina, USA). Raw read data (fastq files) were deposited in the European Nucleotide Archive under the accession number PRJEB40777, and processed with the MTBseq pipeline as described previously [17]. Details on the phylogenetic analysis, molecular cluster definitions, and genotypic drug resistance prediction can be found in the Additional file 1.

### Statistics

A variance analysis to compare proportions of drug resistances from 2012 to 2015 was performed with a Kruskal–Wallis test and a pairwise Dunn’s post-hoc test, considering a significance level of 0.05. Proportions of resistance to individual drugs and 95% confidence intervals (CI) were calculated with the Wilson Score interval. We employed univariate and multiple logistic regression models to obtain odds ratios for factors associated with recent transmission, i.e. molecular clusters, by using R version 4.0.2 and the glm function. Response variables were transformed to 0 (ungrouped) and 1 (clustered). As predictors for an increased transmission likelihood, we analyzed the variables MTBC lineage, number of genotypic resistances, age, gender, HIV status, and genotypic resistance to isoniazid, ethambutol, pyrazinamide, and prothionamide. Other drug resistances were found at very low prevalence throughout the study period and were not considered as predictor variables. Most people included in this analysis had a history of TB treatment (84%), so treatment history was also not included as a predictor variable. Missing data (NA) was kept as a separate category, and categorical data was transformed to factors. Factors with *P* < 0.1 in the univariate model were included in the multiple logistic regression. The best model was determined with a backwards exclusion approach aiming for the best statistical support, i.e. lowest Akaike information criterion (AIC) value. The resulting model was supported by a forward selection approach. Differences between patient characteristics (included versus excluded patients) were determined with chi-squared tests for categorical variables, and with a Mann Whitney U test for age. For chi-squared tests, we treated missing data as its own category, and in addition performed pairwise Fisher exact tests, excluding missing data.

## Results

### Patient characteristics and treatment outcomes

Specimens from 261 patients with RR/MDR-TB were received at the laboratory during the study period. Out of these 195 (75%) were included in the current analysis. For 66 patients, WGS data was not available because specimens could not be either cultured or sub-cultured, or extracted DNA was not suitable for WGS. These 66 excluded patients had similar age, sex, and geographic distribution as those included in the analysis; there were more excluded patients with an unknown HIV status and history of TB treatment relapse (Additional file 4: Table S3). Of the 195 patients with specimens included in the analysis, the median age was 34 years (IQR 27–43 years), 78 (40%) were female, and 163 (84%) had a known history of TB treatment. In total, 149/195 (76.4%) patients started MDR-TB treatment in the study period, including 126/149 (84.6%) patients with a good MDR-TB therapy outcome, i.e. cured or treatment completed, 15/149 (10.1%) patients who died after starting MDR-TB treatment, 5/149 (3.4%) patients who were lost to follow-up, and 3/149 (2.0%) patients who failed the treatment. The remaining 46/195 (23.6%) patients did not start MDR-TB treatment and were lost to follow-up.

### MTBC population structure in Cameroon

**Fig. 1:**
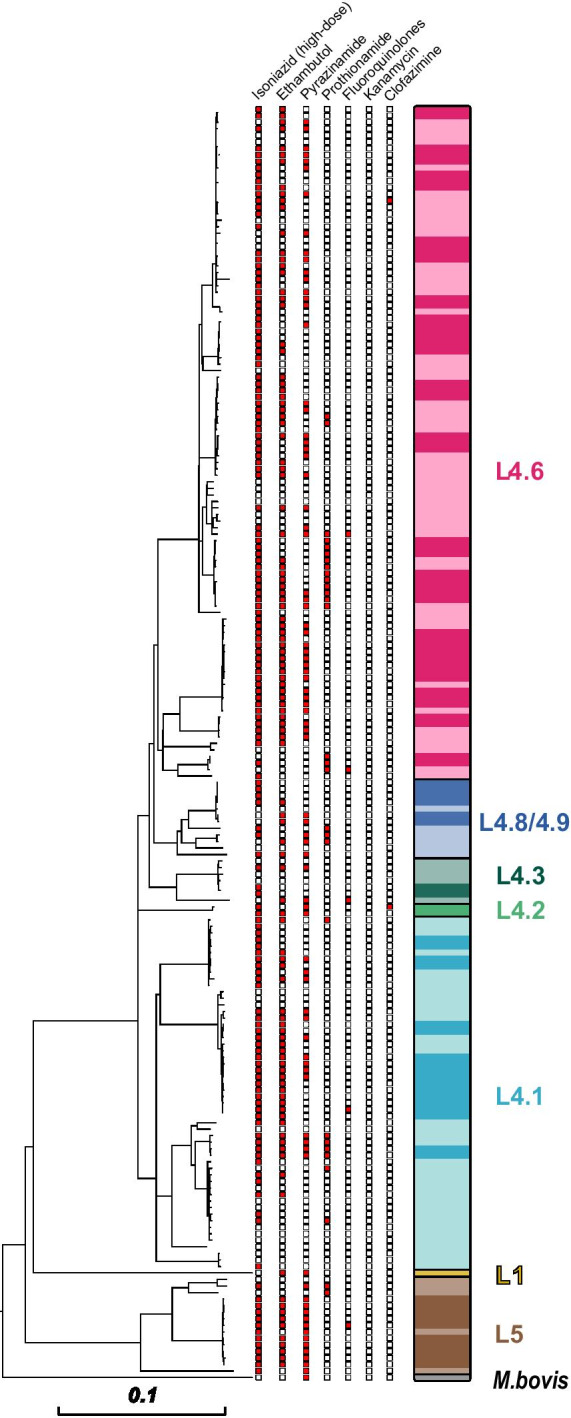
Drug resistance and molecular clusters of multidrug resistant (MDR) *Mycobacterium tuberculosis* complex (MTBC) strains in Cameroon. Maximum likelihood phylogeny of 195 rifampicin resistant and MDR-MTBC strains from Cameroon (2012–2015). MTBC lineages are color coded, stronger colours denote closely related strains (≤ 5 SNP pairwise distance) as surrogate for recently transmitted strains. Genotypic resistances to individual drugs are represented by red squares

Based on 10,838 SNPs, we calculated a maximum likelihood phylogeny using a general time reversible substitution model, and further classified MTBC strains into different phylogenetic lineages according to a recently proposed SNP barcode from Coll et al. [18] (Fig. 1).

In total, 15/195 (7.7%) MTBC strains belonged to lineage 5/*M. africanum*, one strain (0.5%) was classified as lineage 1, one strain (0.5%) was identified as *M. bovis*. The majority, i.e. 178/195 (91.3%), of patients were infected with a lineage 4 MTBC strain. Within lineage 4, we further stratified the strains to particular sub-lineages as follows: 4.1 (54/195, 27.7%), 4.2 (2/195, 1.0%), 4.3 (7/195, 3.6%), 4.6 (103/195, 52.8%), 4.8 (11/195, 5.6%), and 4.9 (1/195, 0.5%) (Fig. 1).

### Genotypic drug resistance profiles

In total, we investigated 27 genes associated with resistance to any drug in the short MDR-TB regimen (Additional file 2: Table S1). With regard to rifampicin resistance, we identified 29 different mutations or combination of mutations mainly in the rifampicin resistance determining region of *rpoB* (Additional file 2: Table S1). Three strains harboured the mutation *rpoB* V170F outside the rifampicin resistance determining region described before as rifampicin resistance marker [19]. Moreover, 57/195 (29.2%) rifampicin resistant strains also had an additional putative compensatory mutation in genes coding for the adjacent RNA-polymerase subunits RpoA or RpoC. We assumed that MTBC strains were resistant to high-dose isoniazid when they harboured a mutation in the catalase gene *katG* at position 315, or a pre-mature stop codon, or a frame shift mutation. Based on this classification, we observed high-dose isoniazid resistance in 77.4% (151/195, 95% CI 71.1–82.7%) of all strains (Table 1).

**Table 1:** Univariate logistic regression analysis to identify factors associated with molecular clusters, i.e. as a surrogate for recent transmission

	Ungrouped (n = 110)	%	Clustered (n = 85)	%	Univariate logistic regression			
					OR	95% lower	95% upper	P value
Age								
> 50	18	16.4	6	7.1	REF			
30–50	51	46.4	44	51.8	2.6	0.9	7.1	0.06
< 30	34	30.9	30	35.3	2.6	0.9	7.5	0.07
Unknown	7	6.4	5	5.9	2.1	0.5	9.4	0.31
Gender								
Female	41	37.3	37	43.5	REF			
Male	66	60	46	54.1	0.8	0.4	1.4	0.39
Unknown	3	2.7	2	2.4	0.7	0.1	4.7	0.75
HIV status								
Negative	50	45.5	51	60	REF			
Positive	33	30	20	23.5	0.6	0.3	1.2	0.13
Unknown	27	24.5	14	16.5	0.5	0.2	1.1	0.08
Lineage								
Others	7	6.4	10	11.8	REF			
L4.X	49	44.5	26	30.6	0.4	0.1	1.1	0.07
L4.6	54	49.1	49	57.6	0.6	0.2	1.8	0.39
Genotypic resistances (n)	1.7 (mean)		2.4 (mean)		1.7	1.3	2.3	**6.64E−05**
					(per unit increase)			
Compensatory mutation								
No	85	77.3	53	62.4	REF			
Yes	25	22.7	32	37.6	2.1	1.1	3.8	**0.02**
High dose isoniazid								
Susceptible	39	35.5	5	5.9	REF			
Resistant	71	64.5	80	94.1	8.8	3.3	23.5	**1.53E−05**
Ethambutol								
Susceptible	62	56.4	22	25.9	REF			
Resistant	48	43.6	63	74.1	3.7	2	6.8	**3.02E−05**
Pyrazinamide								
Susceptible	70	63.6	41	48.2	REF			
Resistant	40	36.4	44	51.8	1.8	1.1	3.3	**0.03**
Prothionamide								
Susceptible	87	79.1	69	81.2	REF			
Resistant	23	20.9	16	18.8	0.9	0.4	1.8	0.72

*P*-values < 0.05 indicated in bold text

Factors with *P* ≤ 0.1 were included in backwards exclusion/forward selection procedures to obtain the best supported multiple logistic regression model, which results are reported in the main text only

*OR* odds ratio

**Fig. 2:**
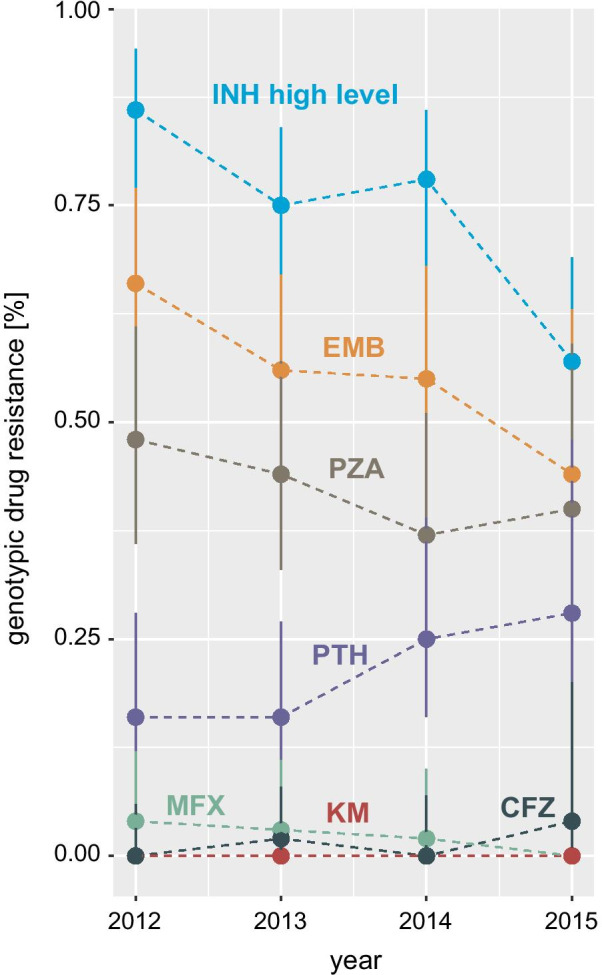
Drug resistance proportions among multidrug resistant (MDR) tuberculosis patients in Cameroon (2012–2015). Annual changes of genotypic drug resistance proportions of 195 rifampicin resistant and MDR *Mycobacterium tuberculosis* complex strains in Bamenda, Cameroon. Colours code for individual drugs, vertical lines represent 95% confidence interval. INH = isoniazid, EMB = ethambutol, PZA = pyrazinamide, PTH = prothionamide, MFX = moxifloxacin, KM = kanamycin, CFZ = clofazimine

Genotypic drug resistance rates to drugs other than isoniazid across all patient were as follows: ethambutol 56.9% (111/195, 95% CI 49.9–63.7%), pyrazinamide 43.1% (84/195, 95% CI 36.3–50.1%), prothionamide 20.0% (39/195, 95% CI 15.0–26.2%), kanamycin 0% (0/195, 95% CI 0.0–1.9%), fluoroquinolones 2.6% (5/195, 95% CI 1.1–5.9%), and clofazimine 1.0% (5/195, 95% CI 0.3–3.7%). We observed overall no differences (0.21 < *P* < 0.92, Kruskal–Wallis test) between individual drug resistance proportions over the 3.5 years study period (Fig. 2).

### MTBC transmission networks

To allocate patients to recent transmission networks, we defined clusters based on a strain-to-strain genetic distance measurement, i.e. a maximum distance of 5 SNPs between at least two strains [20–22].

**Fig. 3:**
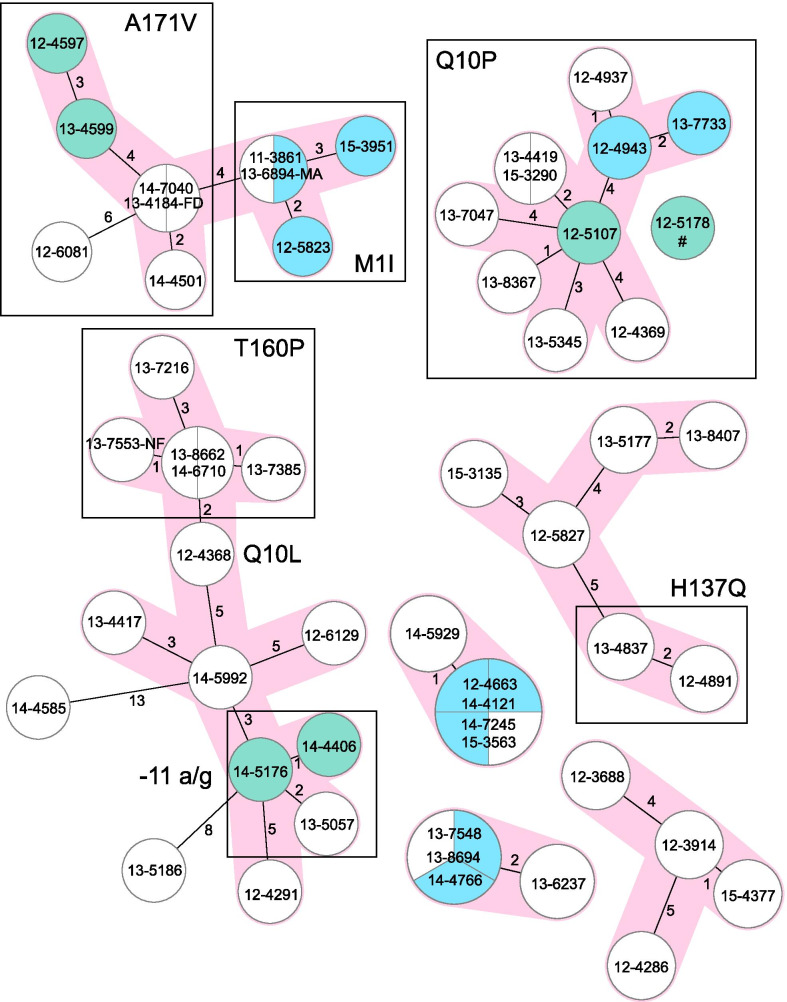
Transmission networks among multidrug resistant (MDR) tuberculosis patients in Cameroon. Seven largest molecular clusters of rifampicin resistant and MDR *Mycobacterium tuberculosis* complex strains in Bamenda, Cameroon. Numbers on connecting lines indicate alelle differences; pink branches connect strains with a maximum distance of five alleles. Boxes indicate strains with identical *pncA* mutations, and identical node colors represent patients with confirmed epidemiological links

Overall, 85/195 (43.6%) strains were grouped in 29 different SNP-based clusters each comprising 2–10 strains. As another measurement of strain relatedness, we performed a core genome multi locus sequence type (cgMLST) analysis based on a gene-to-gene (allele) difference [23]. In total, 89/187 (47.6%) MTBC strains were assigned to a cgMLST clonal complex with a maximum pairwise distance of 5 alleles, mainly confirming the previously defined SNP-based clusters and proportions of strains in clusters. For the cgMLST analysis, 8 strains were excluded with ≥ 10% bad quality alleles (Additional file  2: Table S1). Retrospective contact tracing confirmed that 21/85 (24.7%) patients with strains in SNP-based clusters had an epidemiological link, mainly between household contacts (Additional file 3: Table S2). Confirmed transmission events often involved MTBC strains with identical *pncA* mutations that confer pyrazinamide resistance (Fig. 3, Additional file 3: Table S2). Notably, some SNP-based clusters and cgMLST clonal complexes were further differentiated by distinct *pncA* mutations suggesting recent acquisition of pyrazinamide resistance and continued transmission of pyrazinamide resistant strains (Fig. 3, Additional file 3: Table S2).

### Factors associated with recent transmission of MDR-MTBC strains

We employed logistic regression models to identify bacterial genetic and patient demographic factors associated with recent transmission, using molecular clusters (SNP-based) as a surrogate. In the univariate logistic regression analysis we did not find associations of MTBC lineage, age, gender, or HIV status with recent transmission (Table 1). However, RR/MDR strains with a putative compensatory mutation in genes coding for RNA-polymerase subunits *rpoA*, and *rpoC* were associated with recent transmission (OR 2.1, 95% CI 1.1–3.8, *P* = 0.02). Furthermore, an increasing number of pre-existing resistances against drugs used in the short MDR-TB regimen was associated with an increased odds of patients being recently infected (OR 1.7 per unit increase, i.e. for any additional drug resistance, 95% CI 1.3–2.3, *P* < 0.001). Strains that were considered resistant against high-dose isoniazid (OR 8.8, 95% CI 3.3–23.5, *P* < 0.001), ethambutol (OR 3.7, 95% CI 2.0–6.8, *P* < 0.001), or pyrazinamide (OR 1.8, 95% CI 1.1–3.3, *P* = 0.03) were associated with recent transmission (Table 1).

Next, we considered all predictors with *P* ≤ 0.1 in a multiple logistic regression model and performed a step-wise backwards exclusion of factors aiming for the best supported model, i.e. lowest AIC value. The predictor “number of genotypic drug resistances” showed high collinearity (variance inflation factor 43) and was not considered in the multiple logistic regression model. The best supported model included to against high-dose isoniazid (aOR 7.4, 95% CI 2.6–2.4, *P* < 0.001), resistance to ethambutol (aOR 2.4, 95% CI 1.2–4.8, *P* = 0.014), and an age between 30 and 50 (aOR 3.8, 95% CI 1.3–11.4, *P* = 0.016) as relevant predictors for recent transmission of MDR-MTBC strains. Resistance to pyrazinamide, HIV status, and the presence of a putative compensatory mutation were not found to be strong predictors for recent transmission in the multiple logistic regression analysis. This was confirmed in a forward selection approach.

## Discussion

Our genomic epidemiological study of RR/MDR MTBC strains in Cameroon showed that, besides age of 30–50 years, genotypic resistance to high-dose isoniazid, and ethambutol is a strong predictor for molecular clusters, which is a surrogate for recent transmission. This may be at least partially due to current TB diagnostics and TB patient management in the country. Drug susceptibility tests are prioritized for patients with a high risk of RR/MDR-TB, including patients with a history of TB treatment, prisoners, and known contacts of people with MDR-TB. As a result of this prioritization, RR/MDR-TB patients without these risk factors often start on a first-line drug regimen comprising isoniazid, rifampicin, ethambutol, and pyrazinamide. In 2019 for instance, only an estimated 24% of patients with bacteriologically-confirmed TB in Cameroon had a test for RR/MDR-TB, as compared to the global average of 61% [1]. In fact, undiagnosed RR/MDR-TB has been associated with transmission, as well as selection of additional drug resistances [24], and likely contributes to the association between first-line drug resistances, such as isoniazid and ethambutol, and transmission in our study. Thus, access to universal drug susceptibility testing for all TB patients, as recommended by the WHO [1], is urgently needed in Cameroon to maintain the effectivity of the current MDR-TB treatment regimen, and to curb the evolution of drug resistance.

Although drug resistance proportions remained stable over the relatively short 3.5 year study period, it remains to be determined if pre-existing drug resistances may impact on outcomes of the standardized short MDR-TB regimen in the next years. The very low prevalence of fluoroquinolone, kanamycin and clofazimine resistance likely explains the generally favourable treatment outcomes [16]. However, containing and reducing the risk of RR/MDR-MTBC transmission in general will be crucial to preserve the overall effectiveness of the short MDR-TB regimen and to prevent selection of drug resistances in the region.

There is mounting evidence that globally increasing MDR-TB rates are largely attributable to recent transmission of RR/MDR-MTBC strains, rather than to acquired resistance while on TB treatment [13–15, 25]. Our results suggest that pre-existing drug resistance is an important factor that is associated with recent transmission of RR/MDR MTBC strains in Cameroon, and it should be considered with other factors such as diagnostic delays in many other world settings as it renders initial TB treatment regimens less effective [24, 26]. Patients, especially those with undiagnosed RR/MDR-TB and infected with MTBC strains with additional resistances against ethambutol and pyrazinamide, are more prone to fail initial first-line TB therapies [27]. Likewise, suboptimal therapies and delayed diagnosis increase the time of infectiousness and the likelihood of transmission [27–30].

In addition, bacterial genetic factors such as compensatory evolution, which counteracts the fitness costs of drug-resistance mediating mutations, has also been shown to drive the transmission of MDR-MTBC strains in some settings [14, 24, 31, 32]. In a univariate analysis we found an association of putative compensatory mutations with recent transmission (i.e. molecular clusters), however, including the presence of such mutations did not improve the predictability of recent transmission in the multiple logistic regression analysis.

The observed overall favourable treatment outcomes in our study are in line with a recent phase 3 non-inferiority trial in Bangladesh in patients infected with rifampicin resistant strains but susceptible to fluoroquinolones and aminoglycosides [11].

On the other hand, the short MDR-TB regimen also generated concerns about its efficacy in settings with high resistance rates to ethambutol and pyrazinamide, e.g. Eastern Europe, but also Sub-Saharan Africa, exposing patients to drugs with a high likelihood of pre-existing resistance [6–9, 33]. Thus, decision makers in many settings are confronted with the dilemma that the short MDR-TB regimen is at least non-inferior to a long-term conventional individualized therapy with high cure rates, but it may increase the risk of drug resistance evolution over time. In fact, using drugs despite proven resistance is highly associated with poor treatment outcomes for conventional MDR-TB regimens [24]. Critically, the loss of fluoroquinolones as an effective second-line TB drug would jeopardize the efficacy of both short and conventional MDR-TB therapies [34–36].

Our genomic epidemiological study has particular limitations. The large number of patients lost to follow-up prevented the analysis of factors associated with treatment outcomes. As mentioned before, previously treated patients are prioritized for drug susceptibility testing, and thus are overrepresented in our patient cohort (84% of those studied here had a history of TB treatment). The proportions of pre-existing drug resistances and their implication on the transmission of MTBC strains may not be representative for undiagnosed RR/MDR-TB patients in Cameroon. Furthermore, transmission events that occurred before the study and transmission events during the study including patients that were diagnosed after the study period could not be captured with our analysis. Lastly, the impact of socioeconomic factors on the transmission of MTBC strains could only be partially investigated through retrospective tracing of epidemiological links in molecular clusters, and other relevant factors such as time to diagnosis and detailed treatment histories were not available for inclusion in the analysis.

## Conclusions

We show that resistance to high-dose isoniazid, and ethambutol is associated with recent transmission of MDR-MTBC in Cameroon. Our findings highlight the importance of universal drug susceptibility testing for the early identification of patients who need RR/MDR-TB treatment. The rapid initiation of appropriate treatment regimens will be crucial for both improving treatment outcomes and minimizing transmission of MDR-TB strains as a basis for future MDR-TB control.

## Supplementary Information

**Additional file 1.** Supplementary Methods.

**Additional file 2: Table S1.** Molecular and phenotypic data of MTBC isolates from 195 RR/MDR-TB patients in Cameroon (2013–2015) including patient characteristics.

**Additional file 3: Table S2.** Contact tracing data of patients in molecular clusters and pyrazinamide resistance conferring mutations in pncA.

**Additional file 4: Table S3.** Comparison of patient characteristics, included versus excluded RR/MDR-TB patients.

## Data Availability

Raw sequencing data (fastq files) of *Mycobacterium tuberculosis* complex strains were deposited in the European Nucleotide Archive under the Accession Number PRJEB40777.

## Abbreviations

MTBC: *Mycobacterium tuberculosis* complex
RR: Rifampicin resistance
MDR: Multidrug resistance
TB: Tuberculosis
SNP: Single nucleotide polymnorphism
HIV: Human immunodeficiency virus
cgMLST: Core genome multi locus sequence typing
