# Supplementary material for: Transmission patterns of rifampicin resistant Mycobacterium tuberculosis complex strains in Cameroon: a genomic epidemiological study
Source: BMC Infect Dis. 2021 Aug 31;21:891. doi: 10.1186/s12879-021-06593-8 (PMC8406724; doi:10.1186/s12879-021-06593-8)
Supplement: Supplementary file 1 — Additional file 1. Supplementary Methods. [file 12879_2021_6593_MOESM1_ESM.docx]

**Additional Methods**

**Routine phenotypic and molecular drug susceptibility tests**

Routine diagnostics were performed at the TBRL Bamenda, a laboratory accredited in accordance with the recognized International Standard ISO 15189:2012 (SANAS Accredited Medical Laboratory, No. M0593). Rifampicin resistance was detected either by Xpert MTB/RIF (Cepheid, U.S.) or Genotype MTBDR*plus* (Hain Life Science, Germany) according to the manufacturer’s instructions. Sputum specimens from patients with genotypic resistance against rifampicin and specimens from patients with smear-positive TB to be subjected to routine drug susceptibility testing were decontaminated using the N-acetyl-L-cysteine–NaOH (NALC-NaOH) method. Culture confirmation and cultivation was performed on both mycobacterial growth indicator tubes (MGIT) using the BACTEC MGIT 960 system (BD, U.S.) and Löwenstein-Jensen solid media. Following WHO guidelines, any specimen with genotypic resistance against isoniazid and/or rifampicin resistance as detected by either Xpert MTB/RIF or MTBDR*plus* was phenotypically tested using the proportion method on Löwenstein-Jensen media for isoniazid and/or rifampin resistance, with critical concentrations of 0.2 mg/L and 40 mg/L respectively [1][2]. All rifampicin-resistant MTBC strains were further screened for phenotypic resistance to second-line TB drugs, i.e. kanamycin (30 µg/mL) and ofloxacin (2 µg/mL), using the proportion method. From strains on LJ media or MGIT, genomic DNA was extracted using a CTAB method as described previously [3]. The extracted DNA was sent to the Research Center Borstel for further analysis.

**Next generation sequencing**

WGS was performed on an Illumina NextSeq 500 instrument using Nextera XT library preparation kit according to manufactures instructions (Illumina, USA). Raw read data (fastq files) were deposited in the European Nucleotide Archive under the accession number PRJEB40777, mapped to the *M. tuberculosis* H37Rv genome (NC_000962.3) using BWA-MEM [4], and mappings were refined with the GATK software package [5]. All datasets had a minimum mean genome wide coverage of at least 50-fold. Variants (single nucleotide polymorphisms (SNPs), insertions and deletions (InDels)) were detected with the MTBseq pipeline as described previously [6]. Briefly, variants were called with a minimum coverage of 4 reads in both forward and reverse orientation, 4 reads calling the allele with at least a Phred score of 20, and 75% allele frequency. Multiple consecutive SNP calls (in a 12 bp window), that could reflect artificial variant calls around InDels, drug resistance associated genes, and repetitive regions (e.g. PPE/PGRS genes), were excluded. All remaining SNPs that matched the above-mentioned threshold levels in at least 95% of all strains were considered as valid and used for a concatenated sequence alignment.

**Phylogenetic reconstruction**

A maximum likelihood (ML) tree was calculated with FastTree 2.1.9 (double precision for short branch lengths) [7] using a general time reversible (GTR) nucleotide substitution model and 1,000 resamples to infer bootstrap values for internal branches. The consensus tree was rooted with the “midpoint root” option in FigTree [8] and nodes were arranged in increasing order. Phylogenetic lineages (MTBC lineages and known Beijing subgroups) were inferred from specific SNPs based on Coll et al 2014 and Merker et al [9, 10].

**Genome based clusters**

We assigned strains to molecular clusters by considering a maximum pairwise genetic distance between at least two MTBC strains of ≤5 SNPs as surrogate marker for recent transmission events, as suggested by Walker et al [11, 12]. Further, we employed a core genome multi locus sequence type (cgMLST) approach using Ridom SeqSphere+ as described previously [13, 14]. Briefly, we considered all datasets with less than 10% missing/failed allele calls, and strains with a maximum allele distance of 5 were assigned to clonal complexes as suggested in previous studies [14–16].

**NGS-based drug resistance prediction**

We extracted polymorphisms from 27 drug resistance associated genes that are involved in drug resistance mechanisms towards any of the drugs included in the short MDR-TB regimen and three known compensatory target genes (*rpoA*, *rpoC, and ahpC* [17, 18]) (Table S1). Resistance genotypes were defined as follows: in case of wild type alleles (H37Rv reference sequence or synonymous (silent) mutations) for all analyzed resistance associated genes for an individual antibiotic, we inferred antibiotic susceptibility (or genotypic wild type, gWT). Strains with unknown mutations were classified as ‘genotypic non wildtype’ (nonWT), and included in the susceptible group for univariate and multivariate logistic regression analysis. Strains with known resistance markers (see Table S1) were considered as resistant to the respective antibiotic.

**Data collection**

For patients that were identified as being part of a molecular cluster (Table S2), standard investigation questionnaires were completed to identify common transmission settings and epidemiological links. Patients in clusters were contacted and asked to provide informed consent and participate in an interview. The questionnaires were adapted from those used previously (<https://www.gov.uk/government/publications/tb-strain-typing-and-cluster-investigation-handbook>). The questionnaires were completed with information from the patient file and from the interviews when possible. Information collection included previous addresses, household and close contacts, travel and contact with those from abroad, and attendance in congregate settings (e.g. educational, workplace, place of worship, treatment centers, detention, socializing). Transmission links were considered as confirmed if clustered patients named each other or were in the same household; links were considered possible if clustered patients identified as being in the same setting at the same time (e.g. community or hospital).

**References**

1. Hillemann D, Rüsch-Gerdes S, Richter E. Evaluation of the GenoType MTBDRplus assay for rifampin and isoniazid susceptibility testing of Mycobacterium tuberculosis strains and clinical specimens. *J. Clin. Microbiol.* 2007; 45: 2635–2640.

2. Rieder HL, Van Deun A, Kam KM, Kim SJ, Chonde TM, Trebucq A, Urbanczik R. Priorities for Tuberculosis Bacteriology Services in Low-Income Countries. Second. Tuberculosis International Union Against Tuberculosis and Lung Disease; 2007.

3. van Soolingen D, Hermans PW, de Haas PE, Soll DR, van Embden JD. Occurrence and stability of insertion sequences in Mycobacterium tuberculosis complex strains: evaluation of an insertion sequence-dependent DNA polymorphism as a tool in the epidemiology of tuberculosis. *J. Clin. Microbiol.* 1991; 29: 2578–2586.

4. Li H, Durbin R. Fast and accurate short read alignment with Burrows-Wheeler transform. *Bioinforma. Oxf. Engl.* 2009; 25: 1754–1760.

5. McKenna A, Hanna M, Banks E, Sivachenko A, Cibulskis K, Kernytsky A, Garimella K, Altshuler D, Gabriel S, Daly M, DePristo MA. The Genome Analysis Toolkit: a MapReduce framework for analyzing next-generation DNA sequencing data. *Genome Res.* 2010; 20: 1297–1303.

6. MTBseq: a comprehensive pipeline for whole genome sequence analysis of Mycobacterium tuberculosis complex isolates [PeerJ] [Internet]. [cited 2020 Jul 21].Available from: https://peerj.com/articles/5895/.

7. Price MN, Dehal PS, Arkin AP. FastTree 2 – Approximately Maximum-Likelihood Trees for Large Alignments. *PLOS ONE* 2010; 5: e9490.

8. FigTree [Internet]. [cited 2013 Oct 23].Available from: http://tree.bio.ed.ac.uk/software/figtree/.

9. Coll F, McNerney R, Guerra-Assunção JA, Glynn JR, Perdigão J, Viveiros M, Portugal I, Pain A, Martin N, Clark TG. A robust SNP barcode for typing Mycobacterium tuberculosis complex strains. *Nat. Commun.* 2014; 5: 4812.

10. Merker M, Blin C, Mona S, Duforet-Frebourg N, Lecher S, Willery E, Blum MGB, Rüsch-Gerdes S, Mokrousov I, Aleksic E, Allix-Béguec C, Antierens A, Augustynowicz-Kopeć E, Ballif M, Barletta F, Beck HP, Barry CE, Bonnet M, Borroni E, Campos-Herrero I, Cirillo D, Cox H, Crowe S, Crudu V, Diel R, Drobniewski F, Fauville-Dufaux M, Gagneux S, Ghebremichael S, Hanekom M, et al. Evolutionary history and global spread of the Mycobacterium tuberculosis Beijing lineage. *Nat. Genet.* 2015; 47: 242–249.

11. Walker TM, Ip CL, Harrell RH, Evans JT, Kapatai G, Dedicoat MJ, Eyre DW, Wilson DJ, Hawkey PM, Crook DW, Parkhill J, Harris D, Walker AS, Bowden R, Monk P, Smith EG, Peto TE. Whole-genome sequencing to delineate Mycobacterium tuberculosis outbreaks: a retrospective observational study. *Lancet Infect. Dis.* 2013; 13: 137–146.

12. Walker TM, Merker M, Knoblauch AM, Helbling P, Schoch OD, van der Werf MJ, Kranzer K, Fiebig L, Kröger S, Haas W, Hoffmann H, Indra A, Egli A, Cirillo DM, Robert J, Rogers TR, Groenheit R, Mengshoel AT, Mathys V, Haanperä M, Soolingen D van, Niemann S, Böttger EC, Keller PM, MDR-TB Cluster Consortium. A cluster of multidrug-resistant Mycobacterium tuberculosis among patients arriving in Europe from the Horn of Africa: a molecular epidemiological study. *Lancet Infect. Dis.* 2018; .

13. Kohl TA, Diel R, Harmsen D, Rothgänger J, Walter KM, Merker M, Weniger T, Niemann S. Whole-genome-based Mycobacterium tuberculosis surveillance: a standardized, portable, and expandable approach. *J. Clin. Microbiol.* 2014; 52: 2479–2486.

14. Kohl TA, Harmsen D, Rothgänger J, Walker T, Diel R, Niemann S. Harmonized Genome Wide Typing of Tubercle Bacilli Using a Web-Based Gene-By-Gene Nomenclature System. *EBioMedicine* 2018; 34: 131–138.

15. Meehan CJ, Moris P, Kohl TA, Pečerska J, Akter S, Merker M, Utpatel C, Beckert P, Gehre F, Lempens P, Stadler T, Kaswa MK, Kühnert D, Niemann S, de Jong BC. The relationship between transmission time and clustering methods in Mycobacterium tuberculosis epidemiology. *EBioMedicine* 2018; 37: 410–416.

16. Jajou R, Kohl TA, Walker T, Norman A, Cirillo DM, Tagliani E, Niemann S, de Neeling A, Lillebaek T, Anthony RM, van Soolingen D. Towards standardisation: comparison of five whole genome sequencing (WGS) analysis pipelines for detection of epidemiologically linked tuberculosis cases. *Euro Surveill. Bull. Eur. Sur Mal. Transm. Eur. Commun. Dis. Bull.* 2019; 24.

17. Sherman DR, Mdluli K, Hickey MJ, Arain TM, Morris SL, Barry CE, Stover CK. Compensatory ahpC Gene Expression in Isoniazid-Resistant Mycobacterium tuberculosis. *Science* 1996; 272: 1641–1643.

18. Comas I, Borrell S, Roetzer A, Rose G, Malla B, Kato-Maeda M, Galagan J, Niemann S, Gagneux S. Whole-genome sequencing of rifampicin-resistant Mycobacterium tuberculosis strains identifies compensatory mutations in RNA polymerase genes. *Nat. Genet.* 2011; 44: 106–110.
