# Supplementary material for: Transmission patterns of rifampicin resistant Mycobacterium tuberculosis complex strains in Cameroon: a genomic epidemiological study
Source: BMC Infect Dis. 2021 Aug 31;21:891. doi: 10.1186/s12879-021-06593-8 (PMC8406724; doi:10.1186/s12879-021-06593-8)
Supplement: Supplementary file 4 — Additional file 4: Table S3. Comparison of patient characteristics, included versus excluded RR/MDR-TB patients. [file 12879_2021_6593_MOESM4_ESM.pdf]

Table S3. Comparision of patient characteristics, included versus excluded RR/MDR-TB patients

	All MDR/RR-TB cases, n (%)	Included in analysis, n (%)	Not included in analysis, n (%)	*P* -value chi-squared all categories	*P* -value pairwise Fisher extact
	N=261	N=195	N=66		
Age (N=240), mean years (IQR)	35 (IQR 27-43)	34 (IQR 27-43	35 (IQR 29-43)	0.3*	NA
Age, unknown	21	12	9		0.07
Sex					
Female	109 (42%)	78 (40%)	31 (47%)	0.6	0.31
Male	145 (56%)	112 (57%)	33 (50%)		
Unknown	7 (3%)	5 (3%)	2 (3%)		NA
HIV status					
Negative	116 (44%)	101 (52%)	15 (23%)	<0.001	0.10
Positive	69 (26%)	53 (27%)	16 (24%)		
Unknown	76 (29%)	41 (21%)	35 (53%)		NA
Type of case					
Contact	14 (5%)	12 (6%)	2 (3%)	0.002	0.53
New	17 (7%)	14 (7%)	3 (5%)		0.77
Failure	102 (39%)	85 (44%)	17 (26%)		0.76
Return LTFU	13 (5%)	8 (4%)	5 (8%)		0.31
Relapse	99 (38%)	67 (34%)	32 (49%)		0.02*
Other/Unknown	16 (6%)	9 (5%)	7 (11%)		NA
Year					
2012	74 (28%)	56 (29%)	18 (27%)	0.8	0.88
2013	88 (34%)	63 (32%)	25 (38%)		0.45
2014	68 (26%)	51 (26%)	17 (26%)		1.0
2015	31 (12%)	25 (13%)	6 (9%)		0.51
Region					
Littoral	172 (66%)	131 (67%)	41 (62%)	0.7	0.46
Northwest	45 (17%)	34 (17%)	11 (17%)		1.0
Southwest	14 (5%)	9 (5%)	5 (8%)		0.35
West	30 (12%)	21 (11%)	9 (14%)		0.51

* Mann Whitney U test
